# Synthesis of C@Ni-Al LDH HSS for efficient U-entrapment from seawater

**DOI:** 10.1038/s41598-019-42252-4

**Published:** 2019-04-09

**Authors:** Xiaoyu Yuan, Chunyue Yin, Yuanyuan Zhang, Zengyue Chen, Yifan Xu, Jun Wang

**Affiliations:** 1 Key Laboratory of Superlight Materials and Surface Technology, Ministry of Education, Harbin Engineering University, Harbin, 150001 China; 2 College of Materials and Chemical Engineering, Heilongjiang Institute of Technology, Harbin, 150050 China; 3 College of Materials Science and Chemical Engineering, Harbin Engineering University, Harbin, 150001 China; 4 Harbin Engineering University Capital Management Co. Ltd, Harbin, 150001 China; 5 Institute of Advanced Marine Materials, Harbin Engineering University, Harbin, 150001 China

## Abstract

In this paper, a double hollow spherical shell composite modified with layered double hydroxide (C@Ni-Al LDH HSS) was fabricated for uranium(VI) (U(VI)) adsorption. Various batch experiments were carried out to investigate the influence of pH, concentration, time and coexistence ion on extraction. The results showed that the adsorption processes of U(VI) onto C@Ni-Al LDH HSS were spontaneous and endothermic and closely followed pseudo-second-order and Langmuir isotherm models. The equilibrium time and maximum adsorption capacity of C@Ni-Al LDH HSS was 360 min and 545.9 mg g^−1^. FT-IR and XPS analyses proved that the adsorption behavior was primarily attributed to the strong interaction between oxygen-containing functional groups and U(VI). Moreover, the extraction of trace U(VI) (μg L^−1^) in artificial and natural seawater was also studied. The results showed that C@Ni-Al LDH HSS provided a promising application for the efficient extraction of U(VI) from seawater.

## Introduction

Various solutions to energy issues that are given through the uptake of U(VI) from natural seawater have recently received considerable attention^1–4^. Although the concentration of U(VI) in seawater is only 3.3 μg L^−1^, the total amount (about 4.5 billion tons) is approximately 1000 times than that of earth^5^. In recent decades, many approaches have been used to extract U(VI) from aqueous solutions, including those of chemical precipitation^6^, ion exchange^7^, membrane filtration^8–10^ and adsorption^11–13^. In these approaches, adsorption has been considered as a reliable approach due to its high efficiency, simplicity, low emissions and low cost^14–16^. However, it still faces challenges in the form of marked selectivity design, high adsorption capacity and the high removal efficiency adsorbent requires for extraction.

Hollow micro/nanomaterials are widely used in the fields of catalysts^17,18^, sensors^19–21^, environmental remediation^22–25^, and lithium ion batteries^26–28^ due to their clear shape, high carrying capacity, low density and large specific surface area. Compare to their solid counterparts, the hollow micro/nanomaterials provide more possibilities for structural and compositional adjustment, which can be conducive to the rational design of new functional materials for many expected applications^29–31^. Hollow carbon spheres (HCS) not only have the properties of a hollow micro/nanomaterial, they are also chemically stable, low in cost, have adjustable porosity and a short diffusion distance^32,33^. The disadvantage of HCS is that they are prone to agglomeration in aqueous solution, which is not conducive to adsorption.

Layered double hydroxides (LDHs) with topological transformation, large interlayer space, moderate chemical stability and high concentration of active sites are suitable U(VI) adsorption materials^34,35^. However, the LDHs nanosheets are stacked together when use as a single adsorbent. In order to solve the problem, LDHs is usually combined with other materials. In previous study, a layered double hydroxide/graphene (rGO@NiAl-LDH) composite was prepared with a sandwich structure, with the adsorption capacity of U(VI) reaching 277.80 mg g^−1^ ^36^. The introduction of rGO solved the inefficiency of pure LDHs in uptaking U(VI).

In this study, a novel flower-like, double-shell hollow C@Ni-Al LDH HSS complex was synthesized through a simple method. The Ni-Al LDH nanosheets were grown neatly onto the surface of the HCS so as to avoid stacking. TEM, XRD, STEM and XPS technologies were used for the characterization of C@Ni-Al LDH HSS. The key factors affecting the extraction of U(VI), such as pH, concentration, temperature and contact time, were exploited. Moreover, for economic reasons, the adsorption-desorption of the adsorbent was investigated five times. Finally, the extraction of trace concentration of U(VI) (μg L^−1^) in artificial and natural seawater was also studied.

## Results and Discussion

### Characterization

**Figure 1:**
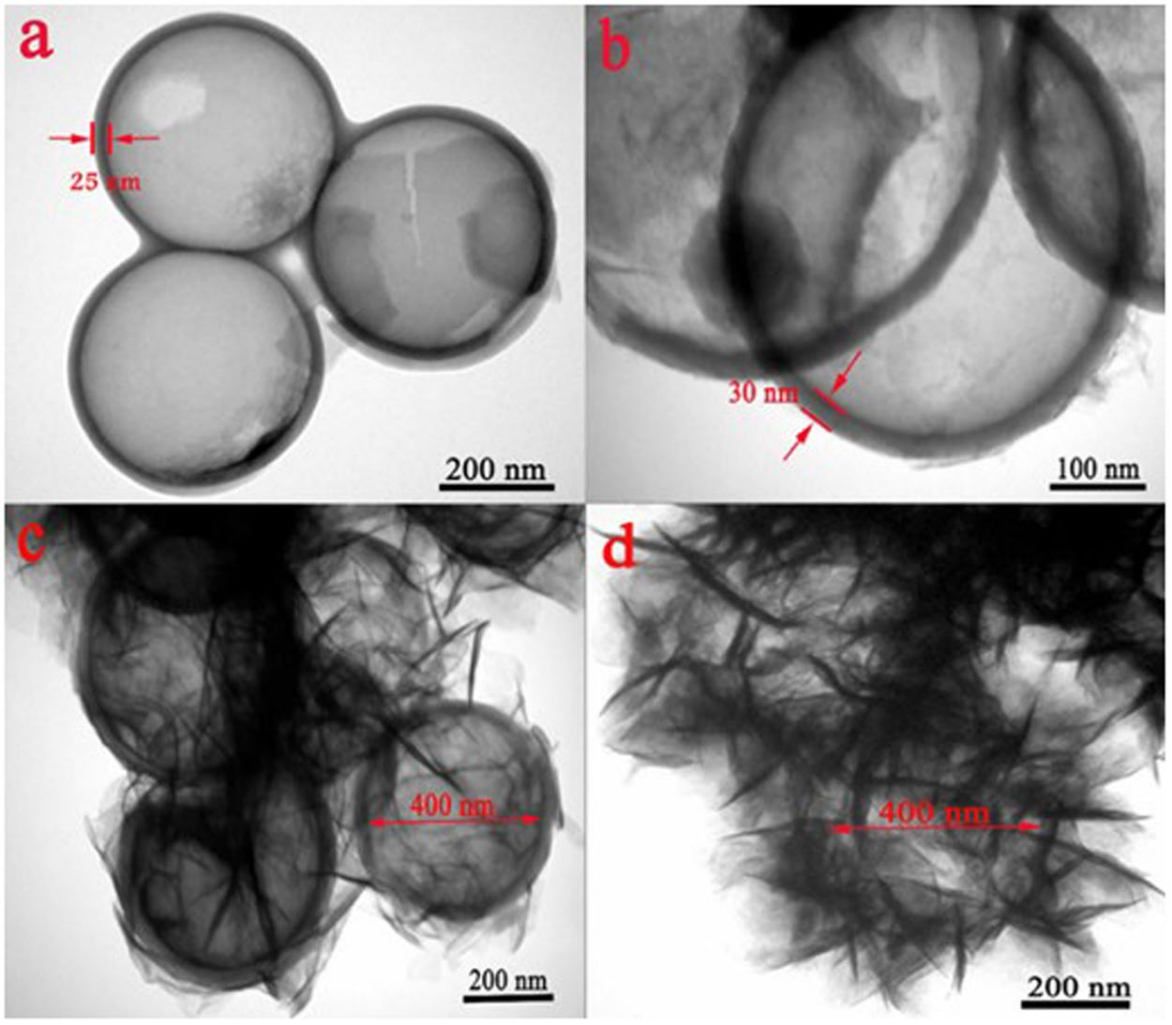
TEM images of HCS (**a**), C@AlOOH HSS (**b**), C@Ni-Al LDH HSS (**c**) and Ni-Al LDH HSS (**d**).

To determine the successful synthesis of C@Ni-Al LDH HSS, TEM measurement was used (Figs 1 and S1). For Fig. S1a, the particle size of the SiO_2_ was approximately 400 nm, with a smooth surface and good dispersibility. After RF (resorcinol-formaldehyde) *in situ* polymerization, the precursor SiO_2_@RF was thicker (54 nm) than that of SiO_2_, indicating that the RF successfully coated the surface of SiO_2_ (Fig. S1b). After calcination, the thickness of the carbon shell of the SiO_2_@C microspheres was 36 nm (Fig. S1c), suggesting that the RF layer shrunk during calcination. HCS showed a hollow spherical shell structure, indicating that SiO_2_ had been successfully removed (Fig. 1a). After AlOOH coating on the HCS surface by a layer by layer deposition process, the thickness of the spherical shell was thickened from 25 to 30 nm (Fig. 1b). Figure 1c shown that the Ni-Al LDH HSS nanosheets uniformly and densely self-assembled on the coordination sites of the HCS shell. After hydrothermal treatment, C@AlOOH HSS was converted into C@Ni-Al LDH HSS double-shell composites. As illustrated in Fig. 1d, the Ni-Al LDH HSS consisted of a large amount of heterogeneous nanoflakes which were superimposed on each other.

**Figure 2:**
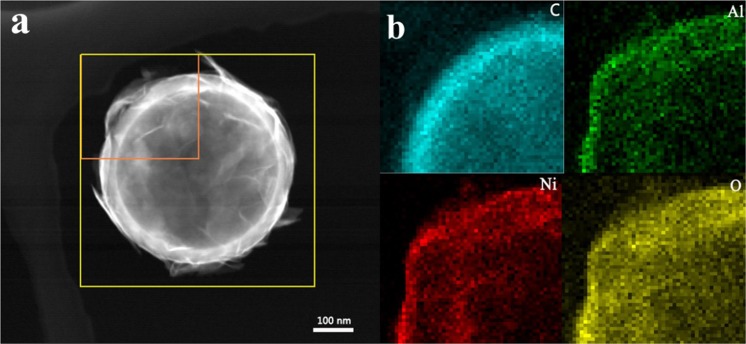
STEM of C@Ni-Al LDH HSS (**a**) and elemental mapping of corresponding C, Al, Ni, and O (**b**).

The double-hollow spherical structure of C@Ni-Al LDH HSS was further confirmed by the aid of STEM and elemental mapping. It was clear that the hollow architecture of C@Ni-Al LDH HSS had an average diameter of 400 nm (Fig. 2a), consistented with the TEM image (Fig. 1c). Further, the elemental mapping (Fig. 2b) clearly verified that the C, Al, Ni, and O elements were homogeneously distributed in C@Ni-Al LDH HSS.

**Figure 3:**
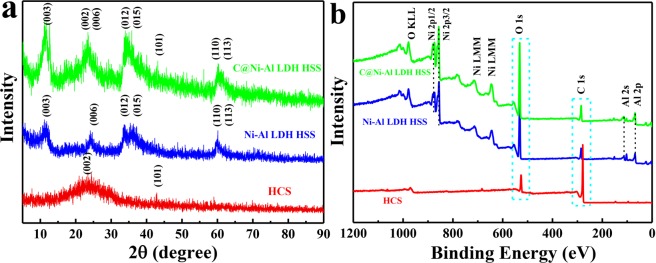
XRD patterns (**a**) and wide scan of XPS spectra (**b**) of HCS, Ni-Al LDH HSS and C@Ni-Al LDH HSS.

As shown in Fig. 3a, for pure Ni-Al LDH HSS, the peaks at 2θ values of 11.52°, 24.16°, 33.66°, 36.16 °, 59.96° and 60.6° could be respectively attributed to the (003), (006), (012), (015), (110) and (113) of the CO_3_^2−^LDH phases^37,38^. The HCS only showed two peaks located at 23.16° and 43.44°, corresponding to the typical graphitic (002) and (101) planes^39–42^ For C@Ni-Al LDH HSS, the (003), (006), (012), (015), (110) and (113) crystal phases of the Ni-Al LDH HSS structure and the (002) and (101) crystallographic planes of the HCS structure were clearly exhibited, indicating that the Ni-Al LDH was successfully assembled on the HCS surface.

The XPS spectra of HCS, Ni-Al LDH HSS and C@Ni-Al LDH HSS were shown in Fig. 3b. Compared with HCS, the new peaks of Ni-Al LDH and C@Ni-Al LDH indicated the appearance of Ni and Al elements in the two HSS. For C@Ni-Al LDH HSS (Fig. S2a), the C 1 s spectra could be divided into C-C, C-O and O-C=O, which located at 284.8, 286.1and 289.1 eV, respectively^43,44^. In the O 1 s spectra of C@Ni-Al LDH HSS (Fig. S2b), the peaks with binding energies of 530.8, 531.7 and 533.3 eV correspond to M-O, -OH and H_2_O, respectively^45,46^.

**Figure 4:**
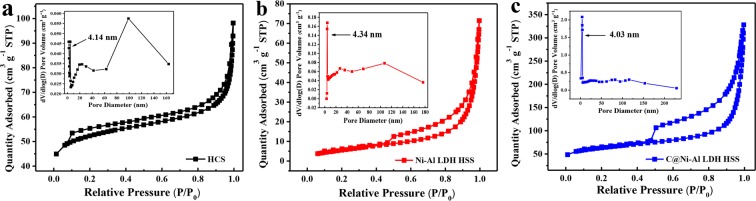
N_2_ adsorption-desorption isotherms and pore-size distribution (inset) of HCS (**a**), Ni-Al LDH HSS (**b**) and C@Ni-Al LDH HSS (**c**).

To further study the detailed structural characteristics of the adsorbent, the N_2_ adsorption-desorption isotherms and the corresponding pore size distributions of HCS, Ni-Al LDH HSS and C@Ni-Al LDH HSS were shown in Fig. 4. All isotherm curves were type IV that possessed a typical H_3_-type hysteresis loop, which suggested that the existence of mesoporous structures^47^. The BET surface area of three samples decreased in the following order: C@Ni-Al LDH HSS (207.69 m^2^ g^−1^) > HCS (166.08 m^2^ g^−1^) > Ni-Al LDH HSS (21.70 m^2^ g^−1^). Concurrently, the respective average pore diameters and pore volumes of each of the above were 17.29 nm and 0.52 cm^3^ g^−1^, 7.36 nm and 0.18 cm^3^ g^−1^, and 17.16 nm and 0.12 cm^3^ g^−1^, respectively. The BJH average pore sizes of the samples were all in the range of 2–50 nm (inset in Fig. 4), further indicating the existence of mesoporous features. Clearly, the C@Ni-Al LDH HSS possessed a larger specific surface area and pore volume than the pure Ni-Al LDH HSS did. In conclusion, the addition of HCS prevented the aggregation of Ni-Al LDH nanosheets, causing more surfaces. Simultaneously, the high surface area of C@Ni-Al LDH HSS furnished more active sites and enhanced the removal of U(VI) ions.

### Effect of initial pH

**Figure 5:**
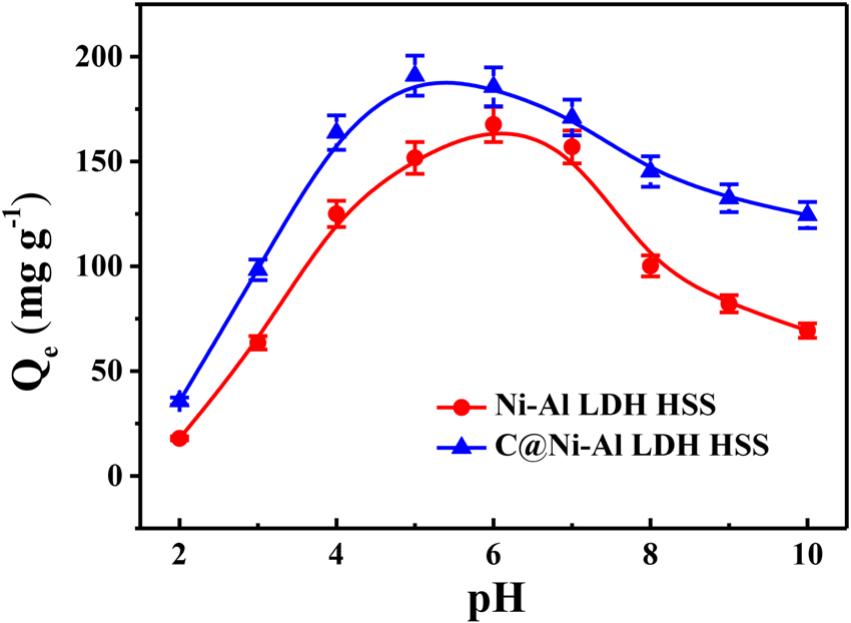
Effect of initial pH on the adsorption capacity (T = 298 K, V = 20 mL, m = 0.01 g, C_0_ = 100 mg L^−1^).

pH played an important role, influencing the distribution of U(VI) species in the solution and the surface properties of the adsorbents. Thus, the impact of the pH upon the U(VI) adsorption behavior of Ni-Al LDH HSS and C@Ni-Al LDH HSS was investigated in the pH range of 2.0–10.0 and shown in Fig. 5. As could be seen, the adsorption capacity of Ni-Al LDH HSS and C@Ni-Al LDH HSS were closely related to pH. For the two materials, the amount of adsorbed U(VI) sharply increased with an increase from pH 2.0–4.0. At a low pH, the poor adsorption was attributed to electrostatic repulsion between U(VI) and the positively charged surface of the protonated adsorbent^48–50^. As the pH increased, the adsorption of U(VI) increased due to the deprotonation of hydroxyl functional groups. At pH 6.0–10.0, the adsorption amount sharply decreased with an increase in pH. This was due to [UO_2_(CO_3_)_2_]^2−^ and [UO_2_(CO_3_)_3_]^4−^ *etc*. predominating in the presence of CO_2_, leading to a reduction in adsorption efficiency^51^.

### Adsorption kinetics

**Figure 6:**
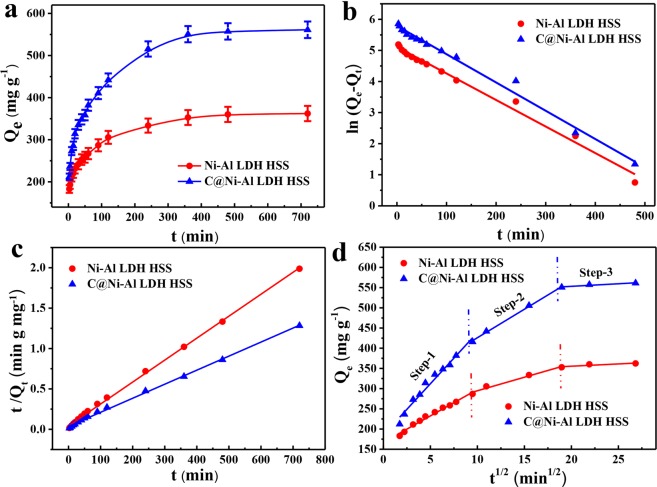
Effect of contact time on U(VI) adsorption capacity (**a**), pseudo-first-order linearly fitted curve (**b**), pseudo-second-order (**c**) and Morris-Weber model (**d**) (T = 298 K, pH = 5, V = 20 mL, m = 0.01 g, C_0_ = 300 mg L^−1^).

To determine the effect of the reaction time on the adsorption kinetics, the change in U(VI) adsorption capacity on materials with contact time was investigated. As illustrated in Fig. 6a, the adsorption capacity of U(VI) on C@Ni-Al LDH HSS rapidly increased with shaking time until the adsorption reached equilibrium at 360 min. Ni-Al LDH HSS reached equilibrium at 480 minutes. The maximum adsorption capacity of the U(VI) of C@Ni-Al LDH HSS (545.9 mg g^−1^) was much higher than that of the U(VI) on Ni-Al LDH HSS (343.2 mg g^−1^), demonstrating that U(VI) in aqueous solution could be effectively removed by C@Ni-Al LDH HSS.

Based on the experimental data, the kinetic processes of Ni-Al LDH HSS and C@Ni-Al LDH HSS were analyzed using pseudo-first-order, pseudo-second-order and Weber-Morris kinetic models (formulas in ESI. 2). As observed in Fig. 6b,c and Table S1, the kinetics of the adsorption of U(VI) by two adsorbents showed a better fit to the pseudo-second-order model (R^2^_Ni-Al LDH HSS_ = 0.9987 and R^2^_C@Ni-Al LDH HSS_ = 0.9976) than to the pseudo-first-order model (R^2^_Ni-Al LDH HSS_ = 0.9879 and R^2^_C@Ni-Al LDH HSS_ = 0.9886). The results were mainly attributed to the chemical action of the U(VI) ions and the functional groups on the surface of the adsorbents. Figure 6d showed the kinetic experiment fitting data for the intra-particle diffusion model on C@Ni-Al LDH HSS and Ni-Al LDH HSS. The adsorption process consisted of three stages for all materials. In the first portion, diffusion occurred from the bulk phase into the pores, with adsorption taking place on the outside surface of the adsorbents^52^. The first-stage straight line corresponded to rapid adsorption in that the instantaneous adsorption could be due to external diffusion. In the second step, the gradual straight line corresponded to the intra-particle diffusion model, which might be ascribed to the loose porous construction of the adsorbents allowing metal ions to diffuse rapidly to the inner surface. Additionally, the second portion of the linear line did not pass through the origin, indicating that intra-particle diffusion was not the sole rate determining factor for controlling U(VI) adsorption. The last straight line showed that the adsorption rate was controlled by the chemical interaction between U(VI) and the effective active site, consistent with the results of the kinetics study^53^. The order of the rate constant k_p_ values, calculated according to the Weber-Morris formula (Eq. S(3)), was as follows: k_p1_ > k_p2_ > k_p3_ (Table S2). Therefore, the adsorption mechanism was regarded as predominantly chemisorption and partly dependent on the pore size of the U(VI) diffusion.

### Adsorption isotherms and thermodynamics

**Figure 7:**
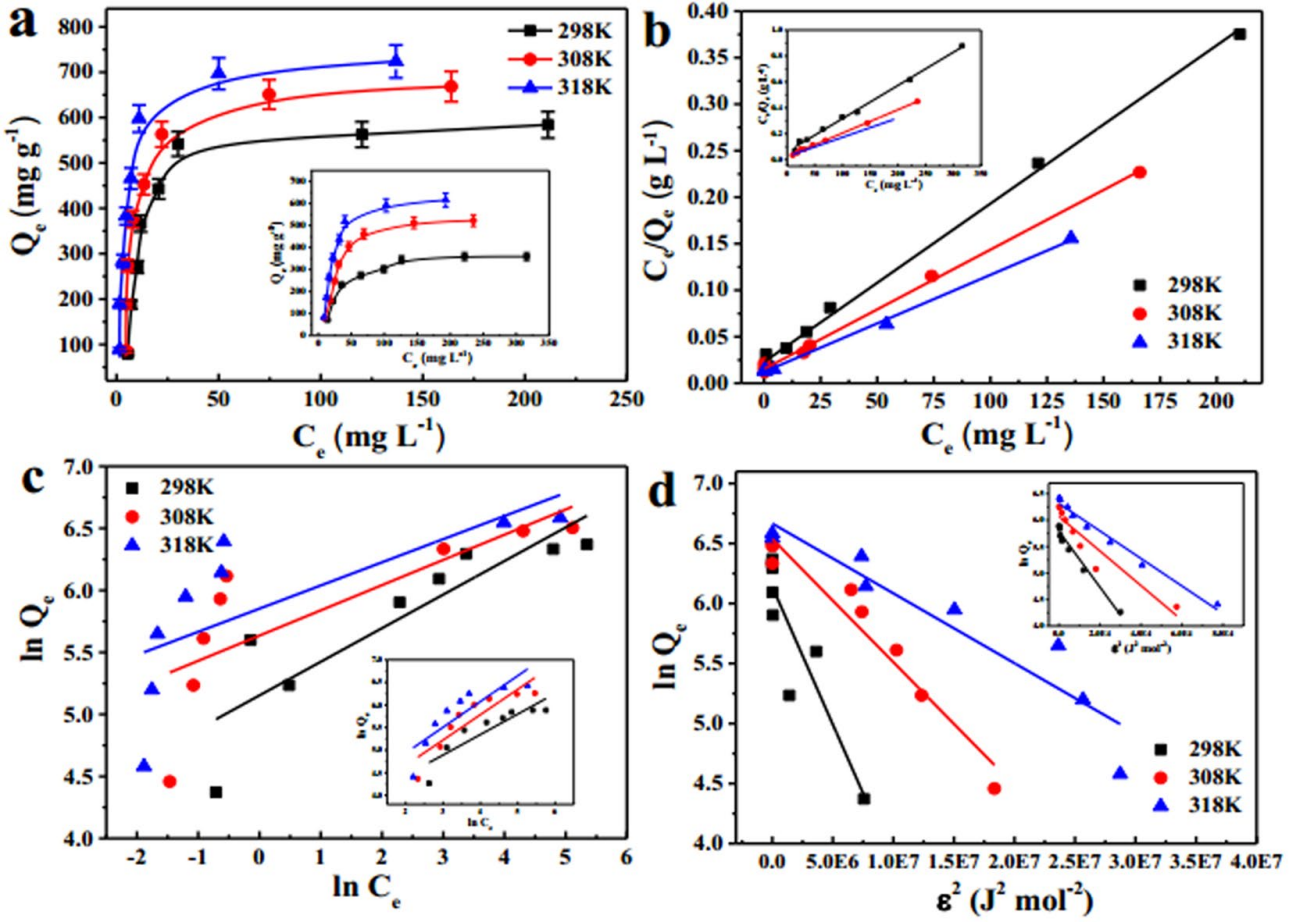
Effect of initial concentration on U(VI) adsorption capacity for C@Ni-Al LDH HSS composites and Ni-Al LDH HSS (inset) (**a**), Langmuir model (**b**), Freundlich model (**c**) and Dubinin-Radushkevich model (**d**) (pH = 5, V = 20 mL, m = 0.01 g, C_0_ = 50–500 mg L^−1^).

The relation between the saturated adsorption amount and the initial concentration of U(VI) was crucial for optimizing the adsorption process. As shown in Fig. 7a, the amount of adsorbed U(VI) on both adsorbents significantly increased with an increase in the initial U(VI) concentration until equilibrium was reached. The maximum adsorption capacity of C@Ni-Al LDH HSS was greater than that of Ni-Al LDH HSS at 298, 308 and 318 K, suggesting that the C@Ni-Al LDH HSS composite provided more active sites to involve U(VI) adsorption. Moreover, the uptake in U(VI) was improved prominently with the increased in temperature, which was potentially due to the diffusion of U(VI) on the surface of the adsorbents being promoted, and the activity of the functional groups on the surface of the adsorbents was enhanced^54^. Three isothermic Langmuir, Freundlich and D-R models^55^ were described as:

1 $$\frac{{{\rm{C}}}_{{\rm{e}}}}{{{\rm{Q}}}_{{\rm{e}}}}=\frac{1}{{{\rm{bQ}}}_{{\rm{m}}}}+\frac{{{\rm{C}}}_{{\rm{e}}}}{{{\rm{Q}}}_{{\rm{m}}}}$$

2 $${{\rm{lnQ}}}_{{\rm{e}}}={\rm{lnk}}+\frac{1}{{\rm{n}}}{{\rm{lnC}}}_{{\rm{e}}}$$

3 $${{\rm{lnQ}}}_{{\rm{e}}}={{\rm{lnQ}}}_{{\rm{DR}}}-{{\rm{\beta }}{\rm{\varepsilon }}}^{2}$$

4 $${\rm{\varepsilon }}=\text{RTln}(1+1/{{\rm{C}}}_{{\rm{e}}})$$

where Q_m_ (mg g^−1^) was the maximum adsorption amount at complete monolayer coverage; C_e_ (mg L^−1^) and Q_e_ (mg g^−1^) were the U(VI) concentration and adsorption capacity at equilibrium, respectively; b was a constant related to the affinity and energy of the adsorbents; k was a Freundlich constant and 1/n was associated with the adsorption intensity; β (mol^2^ J^**−**2^) was the D-R constant related to the adsorption free energy and ε (J mol^−1^) was the Polanyi potential; and T(K) and R (8.314 J mol^−1^ K^−1^) were absolute temperature and gas constants, respectively.

As shown in Fig. 7b,d and Table 1, the simulated results was closer to the Langmuir model (R^2^_C@Ni-Al LDH HSS_ = 0.9966, 0.9983 and 0.9986 and R^2^_Ni-Al LDH HSS_ = 0.9954, 0.9978 and 0.9949) than the Freundlich (R^2^_C@Ni-Al LDH HSS_ = 0.7404, 0.6350 and 0.6366 and R^2^_Ni-Al LDH HSS_ = 0.7640, 0.7563 and 0.6878) and D-R models (R^2^_C@Ni-Al LDH HSS_ = 0.7943, 0.9385 and 0.8744 and R^2^_Ni-Al LDH HSS_ = 0.9670, 0.8764 and 0.9784), suggesting that the adsorption of U(VI) on C@Ni-Al LDH HSS and Ni-Al LDH HSS was monolayer coverage^56^.

**Table 1:** Isotherm parameters for adsorption of U(VI) of Ni-Al LDH HSS and C@Ni-Al LDH HSS.

Materials		Ni-Al LDH HSS			C@Ni-Al LDH HSS		
T		298 K	308 K	318 K	298 K	308 K	318 K
Q_exp,max_		343.2	513.4	615.6	545.9	652.4	695.1
Langmuir	Q_m_	357.8	523.1	628.7	557.2	659.3	701.1
	b	0.0659	0.0463	0.0321	0.1129	1.0635	0.9565
	R^2^	0.9954	0.9978	0.9949	0.9966	0.9983	0.9986
Freundlich	k	34.28	34.53	44.67	172.97	280.15	348.13
	n	2.2081	1.7828	1.7565	3.6911	4.9171	5.3548
	R^2^	0.7640	0.7563	0.6878	0.7404	0.6350	0.6366
Dubinin-Radushkev	Q_DR_	363.9	432.9	572.8	584.7	690.6	785.7
	β	2.29 × 10^−7^	3.26 × 10^−5^	2.60 × 10^−5^	2.29 × 10^−7^	1.03 × 10^−7^	5.82 × 10^−8^
	R^2^	0.9670	0.8764	0.9784	0.7943	0.9385	0.8744

Moreover, the maximum adsorption capacity (Q_exp,max_) of C@Ni-Al LDH HSS for U(VI) attained 545.9 mg g^−1^ at 298 K, 652.4 mg g^−1^ at 308 K, and 695.1 mg g^−1^ at 318 K. The high adsorption capacity was attributed to the active sites provided by HCS and Ni-Al LDH HSS.

**Figure 8:**
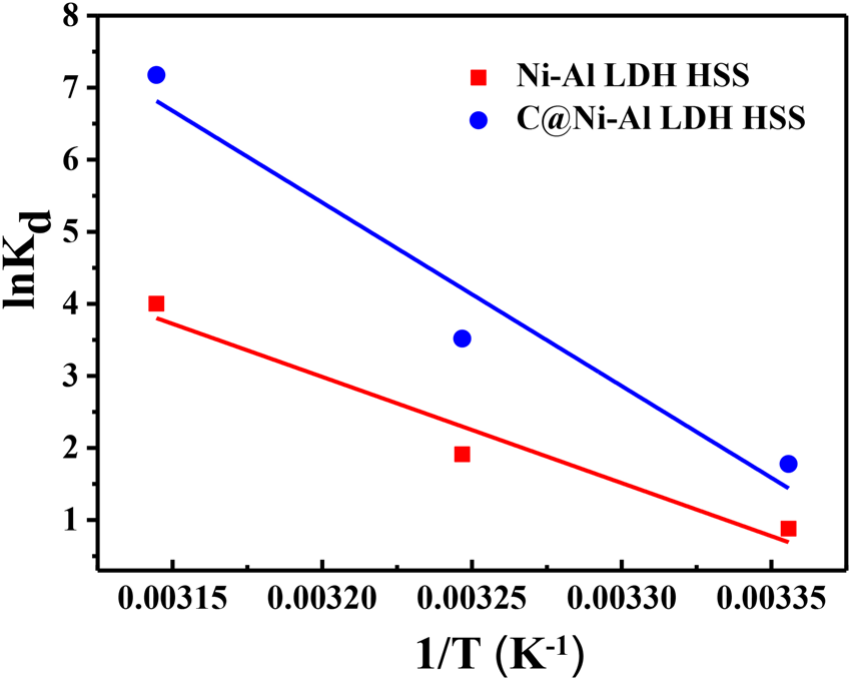
Van’t Hoff plot for uptake of U(VI) by Ni-Al LDH HSS and C@Ni-Al LDH HSS (pH = 5, V = 20 mL, m = 0.01 g, T = 298, 308 and 318 K).

The ΔS^θ^, ΔH^θ^ and ΔG^θ^ thermodynamic parameters were calculated by the following formulas:

5 $${{\rm{lnK}}}_{{\rm{d}}}={{\rm{\Delta }}{\rm{S}}}^{{\rm{\theta }}}/{\rm{R}}-{{\rm{\Delta }}{\rm{H}}}^{{\rm{\theta }}}/{\rm{RT}}$$

6 $${{\rm{\Delta }}{\rm{G}}}^{{\rm{\theta }}}={{\rm{\Delta }}{\rm{H}}}^{{\rm{\theta }}}{-{\rm{T}}{\rm{\Delta }}{\rm{S}}}^{{\rm{\theta }}}$$

where K_d_ was the equilibrium constant (mL g^−1^); T (K) was Kelvin temperature; R (8.314 J mol^−1^ K^−1^) was the gas constant; ΔH^θ^ (kJ mol^−1^) was the standard enthalpy change; ΔS^θ^ (J mol^−1^ K^−1^) was the standard entropy change; and ΔG^θ^ (kJ mol^−1^) was the standard change in Gibbs free energy. The values of ΔH^θ^ and ΔS^θ^ were evaluated from the slope and intercept of the plot of ln Kd *vs*. 1/T (Fig. 8), with the value of ΔG^θ^ calculated using Eq. (6).

From Table 2, the positive value of ΔS^θ^ meaned that the disorder degree of the system increased during U(VI) adsorption, attributable to the structural changed in U(VI) loaded at the solid/solution interface^47^. The negative ΔG^θ^ value and positive ΔH^θ^ value indicated that the processes of U(VI) removal on both absorbents were spontaneous and endothermic^57^. As the temperature increased, the value of ΔG^θ^ became more negative, demonstrating more effective adsorption at higher temperatures. Furthermore, the value of ΔH^θ^ could be used to infer the type of adsorption mechanism. A value of less than 21 kJ mol^−1^ conformed with physical adsorption, whereas the range of 21–418 kJ mol^−1^ conformed with chemical adsorption. That ΔH^θ^_Ni-Al LDH HSS_ = 122.50 kJ mol^−1^ and ΔH^θ^_C@Ni-Al LDH HSS_ = 211.71 kJ mol^−1^ indicate that the adsorption of U(VI) ions is achieved *via* a chemical mechanism.

**Table 2:** The thermodynamic parameters of materials for U(VI) adsorption.

Materials	△H^θ^ (kJ mol^−1^)	△S^θ^ (kJ mol^−1^ K^−1^)	△G^θ^ (kJ mol^−1^)		
			298 K	308 K	318 K
Ni-Al LDH HSS	122.50	0.42	−1.72	−5.88	−10.05
C@Ni-Al LDH HSS	211.71	0.72	−3.57	−10.79	−18.02

### Effect of the co-existing ions

**Figure 9:**
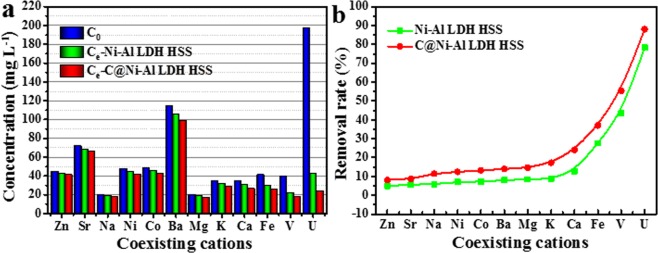
Concentration of different ions before and after adsorption (**a**) and removal rate of U(VI) ions (**b**) (T = 298 K, V = 20 mL, m = 0.01 g).

To evaluate the selectivity of Ni-Al LDH HSS and C@Ni-Al LDH HSS for U(VI) capture, a solution containing 11 kinds of competing metal cations was used (Table S3). As depicted in Fig. 9a,b, the C@Ni-Al LDH HSS composite exhibited better selectivity for U(VI) adsorption than Ni-Al LDH HSS, maintaining a removal rate of up to 90% in the existence of competing ions. Table 3 listed the distribution coefficients (K_d_) of each ions^51^. The values of K^U^_d-C@Ni-Al LDH HSS_ (14866 mL g^−1^) and K^U^_d-Ni-Al LDH HSS_ (7268 mL g^−1^) were markedly higher than the K^M^_d_ values of the other metal ions (M = Zn, Ba, Ca, Mg, Ni, Sr, Co, Fe, Na, V and K). The above-mentioned data indicated that the introduction of HCS improved Ni-Al LDH HSS selectivity and that the C@Ni-Al LDH HSS composite possessed an outstanding affinity towards U(VI) among competing metal ions.

**Table 3:** The selectivity coefficients (K_d_) of various ions.

Ions	Zn	Sr	Na	Ni	Co	Ba	Mg	K	Ca	Fe	V	U
K_d_-_Ni-Al LDH HSS_	105	120	125	153	156	176	184	191	294	763	1538	7268
K_d_-_C@Ni-Al LDH HSS_	178	190	260	284	303	326	345	416	635	1179	2489	14866

### The recyclability of C@Ni-Al LDH HSS composite

**Figure 10:**
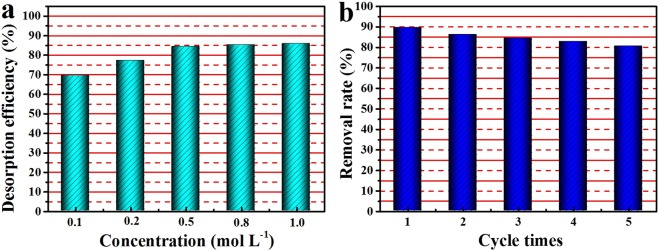
Desorption efficiency for the removal of U(VI) using different concentrations of Na_2_CO_3_ solution (**a**) and removal rate of different cycle times of the C@Ni-Al LDH HSS composites (0.5 mol L^−1^ Na_2_CO_3_) (**b**).

Due to its good adsorption properties, subsequent experiments were carried out with C@Ni-Al LDH HSS. To assess its practicability and regeneration, 0.1–1.0 mol L^−1^ Na_2_CO_3_ eluents on the desorption of U(VI) were carefully investigated, as shown in Fig. 10a. The desorption efficiency was above 80% when Na_2_CO_3_ concentration was greater than 0.5 mol L^−1^, suggesting that Na_2_CO_3_ was an effective eluent for the recovery of the adsorbent. To evaluate the repeatability of the C@Ni-Al LDH HSS composite, the adsorption-desorption experiments with 0.5 mol L^−1^ Na_2_CO_3_ solution were repeated for five cycles. Figure 10b showed the removal rate of C@Ni-Al LDH HSS for five cycles of experiments. The results showed that there was a decrease of only 10% (from 90% to 80%), indicating that C@Ni-Al LDH HSS possessed good reusability for the efficient removal of U(VI).

### Possible mechanism of U(VI) adsorption onto C@Ni-Al LDH HSS

**Figure 11:**
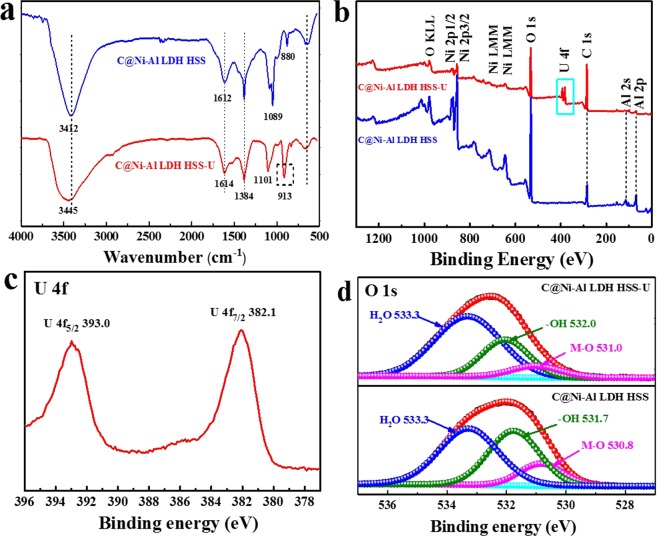
FT-IR spectra (**a**) and XPS spectra (**b**) for C@Ni-Al LDH HSS and C@Ni-Al LDH HSS-U. XPS spectra of U4f (**c**), XPS spectra of O1s for C@Ni-Al LDH HSS and C@Ni-Al LDH HSS-U (**d**).

In order to clarify the interaction mechanism between C@Ni-Al LDH HSS and U(VI), FT-IR and XPS spectroscopies of the C@Ni-Al LDH HSS before and after U(VI) adsorption were recorded (Fig. 11). As can be seen in Fig. 11a, after adsorption, similar peaks of the bands below 800 cm^−1^ slightly red-shifted to 663 cm^−1^, suggesting synergistic effects between U(VI) and metal-oxygen functional groups (M-O). Further, the stretching vibration of the -OH groups red-shift to 3447 cm^−1^, showing that complex (such as [-O⋅⋅⋅H⋅⋅⋅U]^+^ or [-O⋅⋅⋅U]^+^) formations occurred between U(VI) and C@Ni-Al LDH HSS. Importantly, a new peak at 913 cm^**−**1^ corresponded to the antisymmetric stretching vibration of the [O=U=O]^2+^ group, indicating that U(VI) was successfully immobilized^58,59^.

The XPS spectra of C@Ni-Al LDH HSS-U showed clear new double U 4f peaks, when compared with C@Ni-Al LDH HSS (Fig. 11b). As could be seen from the XPS high resolution data in Fig. 11c, strong double U 4f peaks characterized with U 4f_5/2_ (393.0 eV) and U 4f_7/2_ (382.1 eV) in C@Ni-Al LDH HSS-U appear, revealing that U(VI) was captured onto C@Ni-Al LDH HSS^60,61^. As Fig. 11d showed, when compared with the O 1 s spectra of C@Ni-Al LDH HSS before U(VI) sorption, the binding energy of M-O shifted from 530.8 eV to 531.0 eV, and that of -OH shifted from 531.7 and 532.0 eV. The results suggested that the -OH and M-O functional groups interacted with U(VI) ions.

The possible mechanism of U(VI) adsorption was as follows: firstly, due to the 3D double hollow spherical porous shell structure of C@Ni-Al LDH HSS, U(VI) rapidly interacted with the inner and outer surfaces of the adsorbents; then the high surface area and abundant reactive sites contributed to the complexation of functional groups, such as -OH, Al-O and Ni-O, along with the U(VI) and other uranium species, possibly being the reason for the U(VI) adsorption ability of the prepared C@Ni-Al LDH HSS being better than other LDH-based materials (Table 4).

**Table 4:** Comparison of the U(VI) uptake performance of C@Ni-Al LDH HSS with other LDH-based materials (T = 298 K).

Sorbents	Q_max_ (mg g^−1^)	pH	Refs
rGO/LDHs	277.8	4.0	^36^
Ca-Al LDHs	54.8	9.0	^69^
Magnetic Ca-Al LDHs	207.9	6.0	^70^
Fe_3_O_4_@Ni-Al LDHs	26.5	3.0	^71^
C@ Ni-Al LDH HSS	545.9	5.0	This study

### Adsorption experiments in artificial and natural seawater

**Figure 12:**
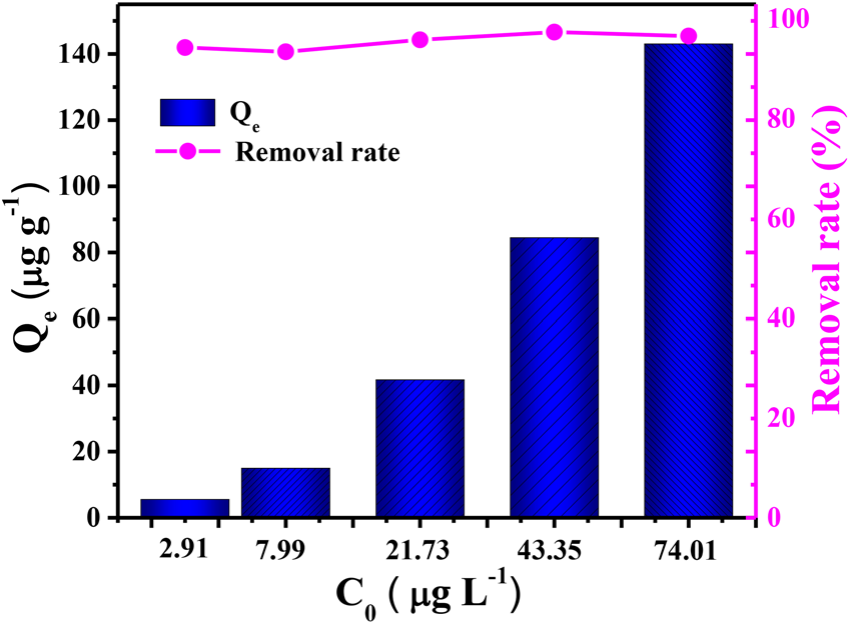
The adsorption of U(VI) by C@Ni-Al LDH HSS in artificial seawater. (T = 298 K, V = 20 mL, m = 0.01 g).

Based on the aforementioned experimental results, the U(VI) adsorption capacity of C@Ni-Al LDH HSS under both artificial and natural seawater was evaluated. The simulated seawater contained a trace concentration of U(VI) and was formulated according to previous reports^45^, possessing a concentration range of 2.91–74.01 μg L^−1^. Fig. 12 showed a high removal rate (more than 90%), implying that C@Ni-Al LDH HSS had potential to effectively extract U(VI) from seawater.

The C@Ni-Al LDH HSS adsorbent was placed in the sea off the coast of Rongcheng, a city in Shandong Province in eastern China. The combination of floating buoys with an anchor made the adsorbent be immersed approximately 3 meters below the surface of the sea stream for 31 days. After 31 days of soaking, the adsorption capacity of C@Ni-Al LDH HSS was 1.24 μg g^−1^. The C@Ni-Al LDH HSS adsorbent in natural seawater before and after the process was shown in Fig. S3.

## Conclusions

In summary, the 3D double hollow spherical shell C@Ni-Al LDH HSS was successfully synthesized and characterized by TEM, STEM, XPS, XRD, and BET techniques. The kinetics data suggested that the processes of adsorption of U(VI) onto C@Ni-Al LDH HSS and Ni-Al LDH HSS fitted well with pseudo-second-order kinetic model. The equilibrium adsorption capacities of C@Ni-Al LDH HSS and Ni-Al LDH HSS were measured and extrapolated using Langmuir, Freundlich and D-R models, with the experimental data found to best fit a Langmuir model. The reusability studies demonstrated that C@Ni-Al LDH HSS possessed high reusability for the efficient removal of U(VI). The adsorption mechanism of C@Ni-Al LDH HSS was mainly attributed to the higher specific surface area of the double hollow spherical shell structure and the strong interaction between abundant oxygen-containing functional groups and U(VI). In addition, the adsorption of U(VI) from artificial seawater and natural seawater was assessed, suggesting that it had potential to efficiently immobilize the radioactive element of U(VI) in seawater.

## Experimental Section

### Materials

Tetraethyl orthosilicate (TEOS) and aluminum isopropoxide (Al(OPr)_3_) were purchased from the Aladdin Chemistry Co. All other chemical reagents were analytical grade and directly used without any additional treatment.

### Synthesis of Ni-Al LDH hollow spherical shell (Ni-Al LDH HSS)

SiO_2_ microspheres were prepared from tetraethoxysilane (TEOS) through an improved Stöber method^62,63^. Ni-Al LDH HSS was obtained as follows^64,65^: (1) 0.2 g as-prepared SiO_2_ microspheres were placed in 20 mL AlOOH sol at room temperature for stirring overnight. The products were centrifuged, washed with ethanol and dried. This process of centrifugation and washing was repeated five times to obtain SiO_2_@AlOOH. (2) A 70 mL aqueous solution containing 0.2 g SiO_2_@AlOOH, 2.9 g Ni(NO_3_)_2_·6H_2_O and 0.3 g urea was transferred in an autoclave at 100 °C for 48 h and then cooled down naturally to room temperature. (3) The targeted resultant Ni-Al LDH HSS was separated by centrifugation, washed with deionized (DI) water and then dried under freezing.

### Synthesis of C@Ni-Al LDH HSS

The intermediate SiO_2_@RF was obtained by SiO_2_ microspheres through a polymer coating process (details in ESI. 5)^66^. The intermediate SiO_2_@C spheres were synthesized by the carbonization of the as-prepared SiO_2_@RF, which were heated at 150 °C for 1 h under N_2_ atmosphere and subsequently at 800 °C for 3 h in the same atmosphere. After etching in 1.0 mol·L^−1^ NaOH for 12 h, the intermediate SiO_2_@C spheres were converted into hollow carbon spheres (HCS). The HCS powder was separated by centrifugation, washed and then dried under freezing.

C@Ni-Al LDH HSS was obtained by *in situ* growth of Ni-Al LDH nanosheets on the surface of HCS by a urea hydrolysis method^67,68^, similar to the preparation method of Ni-Al LDH HSS (details in ESI. 5).

**Figure 13:**
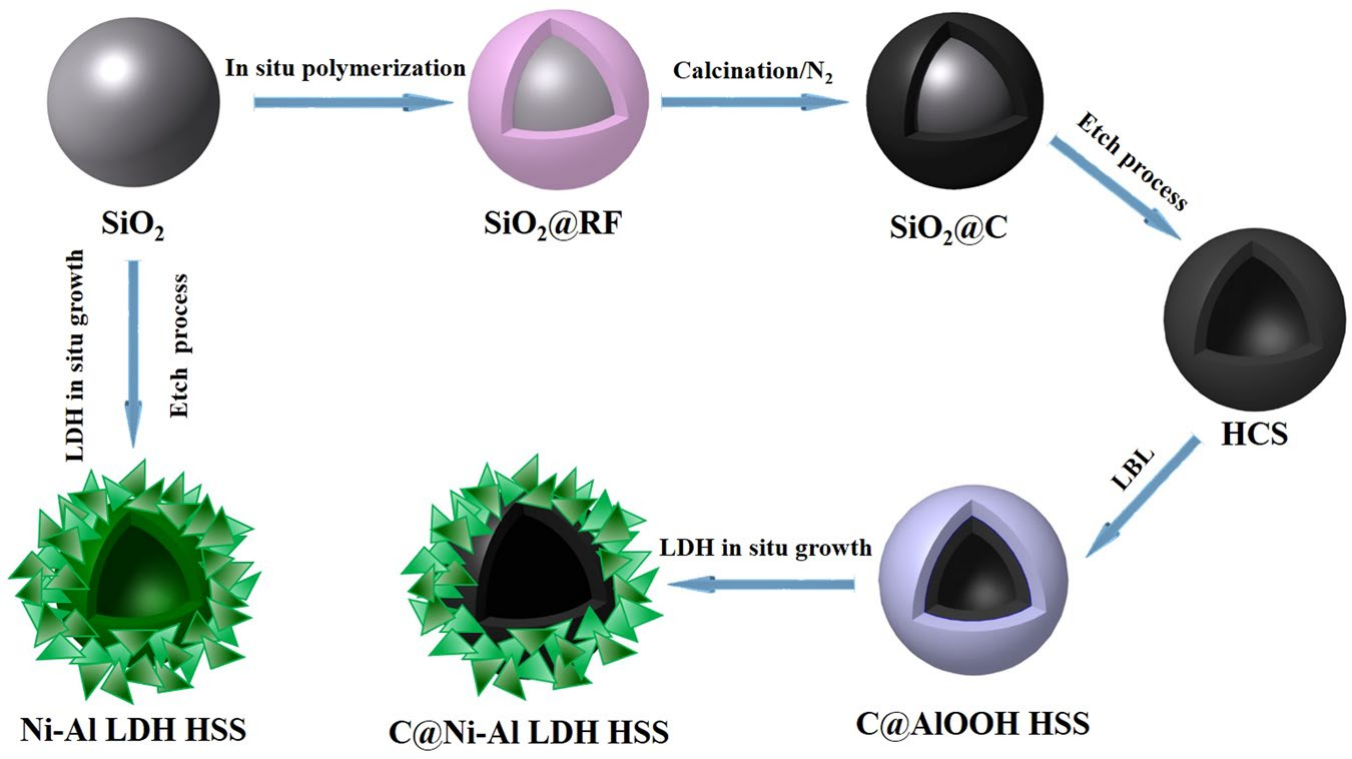
Proposed schematic illustration for the synthesis of Ni-Al LDH HSS and C@ Ni-Al LDH HSS.

The synthetic route of Ni-Al LDH HSS and C@Ni-Al LDH HSS was illustrated in Fig. 13.

### Batch adsorption experiments

2.110 g UO_2_(NO_3_)_2_·6H_2_O was dissolved in 2% nitric acid diluted by DI water, giving a U(VI) concentration of 1000 mg L^−1^. The working U(VI) solutions were prepared by a suitable dilution of the original solution with DI water in the following adsorption experiment. Typically, 10 mg adsorbent was added in the 20 mL UO_2_(NO_3_)_2_·6H_2_O solution with a given concentration and pH, whose initial pH of the solution was adjusted with 0.5 mol L^−1^ Na_2_CO_3_ or HNO_3_. The mixture was shaken for 6 h in a thermostatic shaker bath at the desired temperature. After adsorption, the adsorbent was centrifugally separated and the solution was determined by ICP-AES or ICP-MS. The adsorption capacity Q_e_ (mg·g^−1^) and the removal rate (R%) of U(VI) were calculated using Eqs (7) and (8):

7 $${{\rm{Q}}}_{{\rm{e}}}=\frac{({{\rm{C}}}_{0}-{{\rm{C}}}_{{\rm{e}}})}{{\rm{m}}}\cdot {\rm{V}}$$

8 $${\rm{R}} \% =\frac{({{\rm{C}}}_{0}-{{\rm{C}}}_{{\rm{e}}})}{{{\rm{C}}}_{0}}\times 100 \% $$

where V and m were the volume of the solution (L) and the mass of the adsorbent (g), respectively; C_0_ was the initial U(VI) concentration (mg L^−1^); and C_e_ was the equilibrium U(VI) concentration (mg L^−1^).

### Characterization

Fourier transformation infrared (FT-IR) spectra were conducted with an AVATAR 370, with the XRD patterns recorded on a Rigaku D/max-IIIB diffractometer with Cu Ka irradiation (Ka = 1.54178 Å). The morphological properties of HCS, Ni-Al LDH HSS and C@Ni-Al LDH HSS were investigated by using a transmission electron microscope (TEM, FEI Tecnai G2 S-Twin). XPS spectroscopy measurements were performed by an ESCALAB 250Xi spectrometer with monochromated Al Kα radiation (hν = 1846.6 eV). The specific surface area (S_BET_) and pore size distributions were determined by physical adsorption of N_2_ at −196 °C on an autoadsorption system (Micromeritics ASAP 2010 instrument) and calculated using the Brunauer-Emmet-Teller and Barrett-Joyner-Halenda (BJH) methods.

## Supplementary information

Supplementary information
