# Supplementary material for: Synthesis of C@Ni-Al LDH HSS for efficient U-entrapment from seawater
Source: Sci Rep. 2019 Apr 9;9:5807. doi: 10.1038/s41598-019-42252-4 (PMC6456493; doi:10.1038/s41598-019-42252-4)
Supplement: Supplementary file 1 — Supplementary information [file 41598_2019_42252_MOESM1_ESM.docx]

*Electronic Supplementary Information for*

**Synthesis of C@Ni-Al LDH HSS for efficient U-entrapment from seawater**

*Xiaoyu Yuan* ^1,2,3^***, *Chunyue Yin* ^1,3^*, Yuanyuan Zhang* ^2^, *Zengyue Chen* ^2^, *Yifan Xu* ^2^ *and* *Jun Wang*^1,3,4,5^

*1. Key Laboratory of Superlight Materials and Surface Technology, Ministry of Education, Harbin Engineering University, Harbin 150001, China*

*2. College of Materials and Chemical Engineering, Heilongjiang Institute of Technology, Harbin 150050, China*

*3. College of Materials Science and Chemical Engineering, Harbin Engineering University, Harbin 150001, China*

*4. Harbin Engineering University Capital Management Co. Ltd, Harbin 150001, China*

*5. Institute of Advanced Marine Materials, Harbin Engineering University, 150001, China*

SI.1 **Characterization of adsorbent**

**
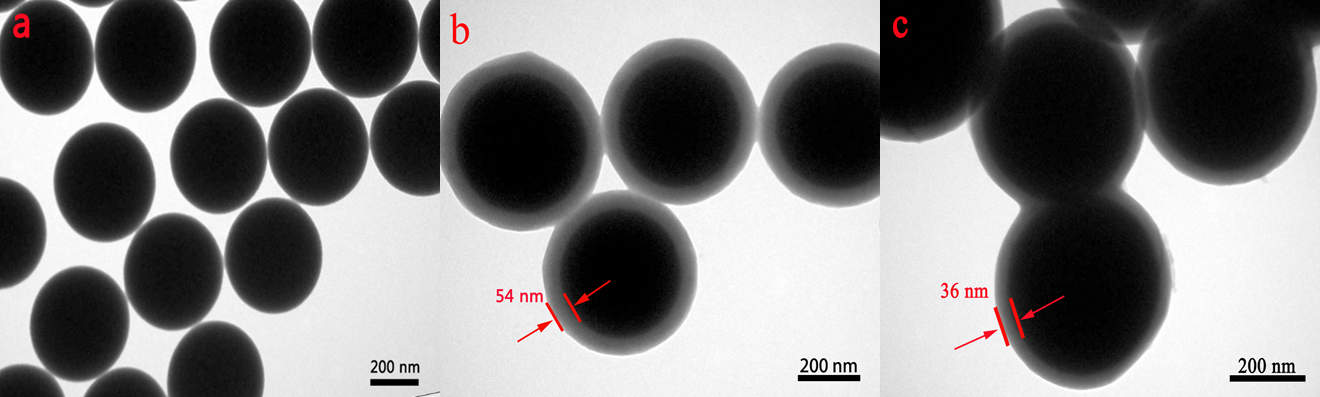
**

**Fig. S1** TEM images of SiO_2_ (a), SiO_2_@RF (b) and SiO_2_@C (c).


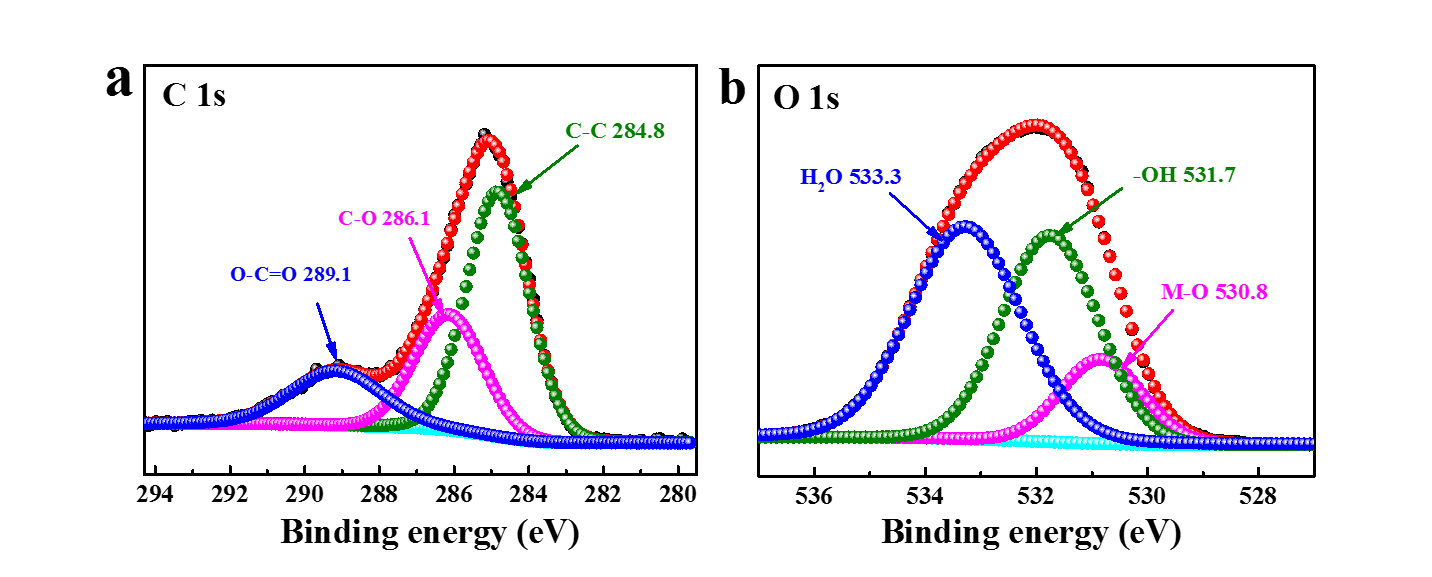


**Fig. S2** High resolution XPS spectra of C1s (a) and O1s (b) for C@Ni-Al LDH HSS before adsorption.

SI.2 Adsorption kinetics

Assuming of the adsorption was controlled by the diffusion step, the pseudo-first-order model kinetic equation expressed as:

$\ln\left( \text{Q}_{\text{e}}\text{-}\text{Q}_{\text{t}} \right)\text{=}\text{lnQ}_{\text{e}}\text{-}\text{k}_{\text{1}}\text{t}$ (1)

Assumption that the adsorption process was based on the chemical adsorption mechanism and the pseudo-second-order model equation was written as:

$\frac{\text{t}}{\text{Q}_{\text{t}}}\text{=}\frac{\text{1}}{\text{k}_{\text{2}}\text{Q}_{\text{e}}^{\text{2}}}\text{+}\frac{\text{t}}{\text{Q}_{\text{e}}}$ (2)

where k_1_ (min^-1^) and k_2_ (mg·g^-1^ min^-1^) were the adsorption rate constants of the pseudo-first-order and pseudo-second-order, respectively. Q_t_ (mg g^-1^) and Q_e_ (mg g^-1^) are the amounts of adsorbed U(VI) at time t (min) and at the sorption equilibrium, respectively.

On the premise of neglecting the liquid film diffusion resistance, the equation based on the Weber-Morris kinetics model was described as follows:

$\text{Q}_{\text{e}}\text{=}\text{k}_{\text{p}}\sqrt{\text{t}}\text{+C}$ (3)

where k_p_ (mg g^-1^ min^-1/2^) was the intra-particle diffusion rate constants, C (mg g^-1^) was a constant describing the boundary-layer effects.

**Table S1** Parameters of pseudo-ﬁrst-order and pseudo-second-order models

| Adsorbents | Pseudo-ﬁrst-order model | | | | Pseudo-second-order model | | | |
| --- | --- | --- | --- | --- | --- | --- | --- | --- |
|  | k_1_ | Q_e, exp_ | Q_e, cal_ | R^2^ | k_2_ | Q_e, exp_ | Q_e, cal_ | R^2^ |
| Ni-Al LDH HSS | 8.5×10^-3^ | 343.2 | 306.01 | 0.9879 | 1.8×10^-4^ | 343.2 | 351.7 | 0.9987 |
| C@Ni-Al LDH HSS | 9.1×10^-3^ | 545.9 | 522.41 | 0.9886 | 8.3×10^-5^ | 545.9 | 558.7 | 0.9976 |

**Table S2** Parameters of intra-particle diffusion kinetics model (T = 298 K)

|  | Materials | Ni-Al LDH HSS | C@Ni-Al LDH HSS |
| --- | --- | --- | --- |
| first stage | kp_1_ | 13.0820 | 25.3303 |
|  | R^2^ | 0.9838 | 0.9733 |
| second stage | kp_2_ | 6.6916 | 14.1023 |
|  | R^2^ | 0.9717 | 0.9969 |
| third stage | kp_3_ | 1.1237 | 1.2735 |
|  | R^2^ | 0.6161 | 0.8259 |

SI.3 **Effect of the co-existing ions.**

The medium for competing ions experiments was deionized water. The original concentration of Sr, Ba, Ca, Ni, Co, Na, Mg, Zn, Fe, K and V was listed in the **Table S3,** which was 1:1 with U (molar rate). “Volume”, “temperature”, “mass” and “time” were 20 mL, 298 K, 0.01 g and 12 h, respectively.

**Table S3**. The original concentrations of the competing ions.

| ions | Ba | Ca | Co | K | Mg | Na | Ni | Sr | Fe | Zn | V |
| --- | --- | --- | --- | --- | --- | --- | --- | --- | --- | --- | --- |
| C_0_  (mg L^-1^) | 114.60 | 34.44 | 48.51 | 34.71 | 19.80 | 19.90 | 47.55 | 71.64 | 41.41 | 44.48 | 38.81 |

SI.4 Adsorption **experiments** in nature seawater


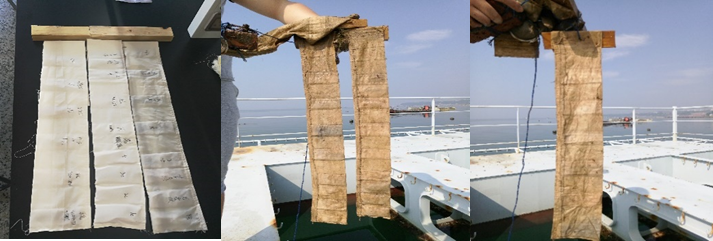


**Fig. S3** The C@Ni-Al LDH HSS adsorbent in the nature seawater before and after the process of extraction.

SI.5 Synthesis of C@Ni-Al LDH HSS

**SiO_2_@RF**. Typically, 0.6 g prepared SiO_2_ spheres was homogeneously dispersed in 53 mL DI water and 21 mL ethanol by ultrasonication. 0.27 g resorcinol, 1.7 g cetyltrimethyl ammonium bromide and 0.1 mL of ammonia (25%-28%) were added to the SiO_2_ suspension in sequence and stirred for 30 min at 35 ^o^C. Subsequently, 0.4 mL formaldehyde was added into the mixture, stirring for 6 h, and aged for 12 h at room temperature. The resulting SiO_2_@RF microspheres were obtained by centrifugation, washing and drying at 60 ^o^C.

**C@Ni-Al LDH HSS**. The AlOOH-coated HCS (C@AlOOH HSS) was prepared by a layer by layer deposition method. First, 0.2 g as-prepared HCS was placed in 20 mL AlOOH sol stirring at room temperature for 12 h. Then, the products were separated by centrifugation, rinsed with ethanol for 2-3 times and dried at room temperature overnight. The described process (agitation, centrifugation, washing, drying) was repeated five times to obtain the C@AlOOH HSS. In a typical synthesis, 0.2 g C@AlOOH HSS was dispersed homogeneously in 70 mL DI water containing 2.91 g Ni(NO_3_)_2_·6H_2_O and 0.3 g urea stirring continued for 30 min. After that, the mixture solution was transferred in a 100 mL autoclave at 100 ^o^C for 2 days, and cooled down naturally to room temperature. Finally, the targeted resultant C@Ni-Al LDH HSS composites were separated by centrifugation, washed several times with ethanol and DI water, and dried at 60 ^o^C.
